# Seizures in Alzheimer’s disease are highly recurrent and associated with a poor disease course

**DOI:** 10.1007/s00415-020-09937-7

**Published:** 2020-06-02

**Authors:** Jonathan Vöglein, Ingrid Ricard, Soheyl Noachtar, Walter A. Kukull, Marianne Dieterich, Johannes Levin, Adrian Danek

**Affiliations:** 1 German Center for Neurodegenerative Diseases (DZNE), Feodor-Lynen-Straße 17, 81377 Munich, Germany; 2 Department of Neurology, Ludwig-Maximilians University, Marchioninistraße 15, 81377 Munich, Germany; 3 Institute for Medical Informatics, Biometry and Epidemiology, Ludwig-Maximilians University, Marchioninistraße 15, 81377 Munich, Germany; 4 Department of Epidemiology, University of Washington School of Public Health, 1959 NE Pacific Street, Seattle, WA 98195 USA; 5 Munich Cluster for Systems Neurology (SyNergy), Feodor-Lynen-Straße 17, 81377 Munich, Germany; 6 German Center for Vertigo and Balance Disorders, Ludwig-Maximilians University, Marchioninistraße 15, 81377 Munich, Germany

**Keywords:** Alzheimer’s disease, Epilepsy, Seizures, Seizure recurrence risk, Seizure prevalence

## Abstract

**Background:**

Seizures are an important comorbidity in Alzheimer’s disease (AD). Conflicting results regarding clinical parameters associated with seizures in AD were previously reported. Data on seizure recurrence risk, a crucial parameter for treatment decisions, are lacking.

**Methods:**

National Alzheimer’s Coordinating Center data were analyzed. Seizure prevalence in AD and an association with disease duration were investigated. Associations of seizures with age of AD onset and with cognitive and functional performance, and seizure recurrence risk were studied.

**Results:**

20,745 individuals were investigated. In AD dementia, seizure recurrence risk was 70.4% within 7.5 months. Seizure history was associated with an earlier age of onset of cognitive symptoms (seizures vs. no seizures: 64.7 vs. 70.4 years; *p* < 0.0001) and worse cognitive and functional performance (mean MMSE score: 16.6 vs. 19.6; mean CDR-sum of boxes score: 9.3 vs. 6.8; *p* < 0.0001; adjusted for disease duration and age). Seizure prevalence increased with duration of AD dementia (standardized OR = 1.55, 95% CI = 1.39–1.73, *p* < 0.0001), rising from 1.51% at 4.8 years to 5.43% at 11 years disease duration. Seizures were more frequent in AD dementia compared to normal controls (active seizures: 1.51% vs. 0.35%, *p* < 0.0001, OR = 4.34, 95% CI = 3.01–6.27; seizure history: 3.14% vs. 1.57%, *p* < 0.0001, OR = 2.03, 95% CI = 1.67–2.46).

**Conclusion:**

Seizures in AD dementia feature an exceptionally high recurrence risk and are associated with a poor course of cognitive symptoms. AD patients are at an increased risk for seizures, particularly in later disease stages. Our findings emphasize a need for seizure history assessment in AD, inform individual therapeutic decisions and underline the necessity of systematic treatment studies of AD-associated epilepsy.

## Introduction

In Alzheimer’s disease (AD), cognitive symptoms represent the intrinsic clinical manifestation, but also non-cognitive symptoms affect individuals with AD [1, 2]. As the prevalence of AD is estimated to further increase in the future, there will be a mounting burden for patients and their families as well as for society [3]. Therefore, non-cognitive symptoms associated with AD will gain importance and will be needed to be addressed. Such a symptom are seizures. An association between Alzheimer’s disease and seizures was reported in several studies with figures for seizure prevalence in AD from 0.5% up to 64% [4, 5]. In autosomal dominant AD, that can be considered as a model disease of the sporadic form without age-associated comorbidities, there is an increased likelihood for the occurrence of seizures [2, 6], even very early in the disease course when cognitive impairment is absent [7]. A causal connection between AD and seizure pathophysiology is suggested by a number of mouse models for AD, which frequently show epileptiform activity on EEG and overt seizures [8]. The National Alzheimer’s Coordinating Center (NACC), established to facilitate collaborative research, runs a database that is unique for its size [9]. The clinical information entered follows the standardized manner of the Uniform Data Set (UDS) [10, 11]. Open questions with respect to the recurrence risk of seizures in AD including potential consecutive treatment implications, regarding the connection between seizures and disease course as well as regarding the association of seizures with disease duration subsist. Using the NACC dataset, we aimed to investigate the clinical course of seizures and possible implications for antiepileptic treatment decisions.

## Methods

### Participants

The NACC database containing data from 34 past and present Alzheimer’s disease centers (ADCs) in the USA was used for this study. Data of the UDS [10] from visits conducted between September 2005 and February 2016 were analyzed. NACC data has been described in detail before [8–12]. Research utilizing the NACC database was approved by the Institutional Review Board of the Ludwig-Maximilians University, Munich, Germany. Informed consent from individuals that are part of the NACC datasets was obtained at the respective ADCs.

### Assessment of seizures and AD stage

In the UDS [10], occurrence of seizures is assessed by information obtained from the study participant and a mandatory co-participant interview, from medical records and from observation. Four options are at choice: “Absent” (seizures are not indicated), “Recent/Active” (seizures happened within the last year or still require active management and are consistent with information obtained from the subject and co-participant interview), “Remote/Inactive” (seizures existed or occurred in the past—more than one year ago—but were resolved or there is no treatment currently under way), or “Unknown” (insufficient information available from the subject and co-participant interview). From now on, if “Seizures” are stated “Recent/Active”, they are referred to as active seizures.

The cognitive status is categorized in the NACC-UDS according to the four mutually exclusive options normal cognition, MCI (mild cognitive impairment), impaired-not-MCI (subjects who are objectively cognitively impaired but who do not meet the criteria for MCI considering the subject’s presentation, test results, symptoms, and clinical evaluation) or dementia. In subjects with any cognitive impairment (dementia, MCI, or impaired-not-MCI), the presumptive etiologic diagnosis is stated by the respective trained rater. For an etiologic diagnosis of AD, either the NINCDS/ADRDA (National Institute of Neurological and Communicative Disorders and Stroke/Alzheimer’s Disease and Related Disorders Association) criteria [13] or the NIA-AA (National Institute on Aging-Alzheimer’s Association) criteria for AD dementia [14] were applied.

**Fig. 1:**
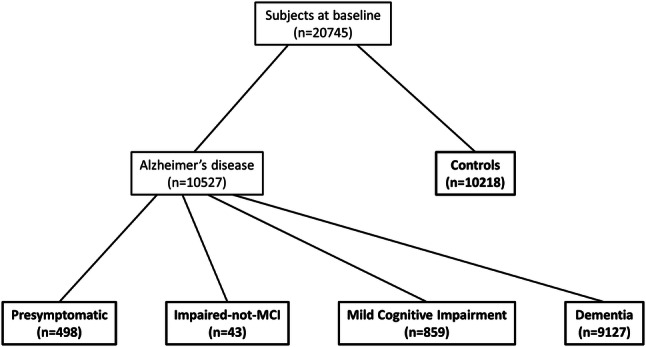
Flow chart depicting the study population at baseline and separation into subgroups. For pre-symptomatic Alzheimer’s disease, a normal cognitive status at baseline and a diagnosis of Alzheimer’s disease dementia at follow-up was required. For impaired-not-MCI due to Alzheimer’s disease and MCI due to Alzheimer’s disease, both a presumptive etiologic diagnosis of Alzheimer’s disease at baseline and a diagnosis of Alzheimer’s disease dementia at follow-up were required. Groups that were included in analyses are shown in bold. *MCI* mild cognitive impairment

For analysis of seizure prevalence at baseline visits, we defined five groups. Presymptomatic AD (normal cognition at baseline), AD impaired-not-MCI, AD MCI, AD dementia, and controls (Fig. 1). The cognitive status of a participant is determined at every NACC study visit with the options “normal cognition”, “impaired-not-MCI”, “MCI” and “dementia” at choice. If a participant was attested “normal cognition” based on the overall impression taking all assessments of the respective visit into account by the respective trained clinical raters at all study visits (at baseline and follow-ups), she or he was allocated to the control group for this study. To increase the accuracy of the etiologic diagnosis of AD in cognitively impaired participants and to define the group with presymptomatic AD, a diagnosis of AD dementia at a subsequent visit of the respective participant was stipulated for the classification into a pre-dementia stage of AD.

### Clinical course of seizures in AD dementia

In individuals with AD dementia at baseline, the prevalence of active seizures and disease duration at the baseline visit and 8 subsequent follow-up visits were calculated. To determine disease duration, each individual’s age of onset of cognitive decline was subtracted from his or her age at the respective visit. The age of onset of cognitive decline is assessed for each participant in a standardized manner as part of UDS.

For assessment of seizure recurrence risk in AD dementia we calculated the proportion of patients with active seizures at the first follow-up visit of those who already suffered from active seizures at baseline and presented for follow-up. Cognitive and functional performance at baseline was compared between AD dementia patients with and without seizures.

### Statistical analyses

Clinical and demographic features of the study population at baseline were analyzed and compared between subgroups using Fisher’s exact test, Kruskal–Wallis test and Mann–Whitney *U* test (Tables 1 and 2). Each AD group (presymptomatic, impaired-not-MCI, MCI, dementia) was compared to controls regarding the prevalence of active seizures and a history of seizures at baseline using Fisher’s exact test (Fig. 4). In cases of statistically significant group differences, odds ratios (OR) and 95% confidence intervals (CI) were calculated. Regarding a potential association between seizure prevalence and disease duration, different transformations of the disease duration were considered to improve the fit between the two variables. The best fit was provided by a logistic regression with disease duration square-transformed and standardized as explanatory variable (Fig. 3). For analysis of respective baseline cognitive and functional performance we compared AD dementia patients with and without seizures with respect to mean mini mental state examination (MMSE) [15] and clinical dementia rating-sum of boxes (CDR-SB) [16] scores in a generalized linear model with disease duration and age as co-variables. To assess the impact of seizures on the cognitive and functional trajectory as measured by CDR-SB, a general linear model with history of seizures (yes vs. no) and disease duration as independent variables and CDR-SB as dependent variable including a history of seizures*disease duration interaction was used. *p* values below 0.05 were considered statistically significant. All tests were performed two-sided. The Statistical Package for the Social Sciences (IBM SPSS Statistics, Version 23) and R (version 3.5.1) were used for statistical analysis.

**Table 1:** Study subpopulations, baseline characteristics and prevalence of seizures

	Pre-symptomatic AD (*n* = 498)	AD impaired-not-MCI (*n* = 43)	AD MCI (*n* = 859)	AD dementia (*n* = 9127)	Controls (*n* = 10,218)	*p* value
Active seizures, No. (%)	2 (0.4)	1 (2.33)	1 (0.12)	138 (1.51)	36 (0.35)	< 0.001^1^
History of seizures, No. (%)	9 (1.8)	1 (2.33)	11 (1.28)	287 (3.14)	161 (1.57)	< 0.001^1^
Mean age ± SD, years	79.8 ± 7.7	74.9 ± 9.0	75.1 ± 8.3	75.1 ± 9.9	69.8 ± 10.9	< 0.001^2^
Female: male, No.	325: 173	23: 20	427: 432	5140: 3987	6730: 3488	< 0.001^3^
Mean MMSE score ± SD	28.2 ± 1.8	27.2 ± 2.6	26.1 ± 2.7	19.5 ± 6.7	28.9 ± 1.4	< 0.001^2^
Mean CDR-SB score ± SD	0.3 ± 0.6	1.7 ± 1.3	1.9 ± 1.2	6.8 ± 4.4	0.1 ± 0.3	< 0.001^2^
Mean global CDR score ± SD	0.10 ± 0.20	0.49 ± 0.17	0.51 ± 0.11	1.19 ± 0.74	0.04 ± 0.14	< 0.001^2^

*AD* Alzheimer’s Disease, *MCI* mild cognitive impairment, *SD* standard deviation, *MMSE* Mini mental state examination, *CDR-SB* clinical dementia rating-sum of boxes

^1^Fisher’s exact test comparing the groups AD dementia and controls. No statistically significant differences were found while comparing each other AD group to controls

^2^Kruskal–Wallis test comparing the groups pre-symptomatic AD, AD impaired-not-MCI, AD MCI, AD dementia and controls

^3^Pearson’s Chi square test comparing the groups pre-symptomatic AD, AD impaired-not-MCI, AD MCI, AD dementia and controls

**Table 2:** Comparison of clinical and demographic baseline characteristic between Alzheimer’s disease dementia patients with and without seizures

	AD dementia patients with seizures (*n* = 287)	AD dementia patients without seizures (*n* = 8840)	Total (*n* = 9127)	*P* value
Mean age ± SD, years	71.1 ± 10.9	75.2 ± 9.8	75.1 ± 9.9	< 0.001^1^
Female: male, No	154: 133	4986: 3854	5140: 3987	0.36^2^
Mean age of onset ± SD, years	64.7 ± 11.2	70.4 ± 9.9	70.2 ± 10.0	< 0.001^1^
Mean disease duration ± SD, years	6.3 ± 4.1	4.8 ± 3.3	4.8 ± 3.4	< 0.001^1^
Mean MMSE score ± SD	16.6 ± 8.6	19.6 ± 6.6	19.5 ± 6.7	< 0.001^3^
Mean CDR-SB score ± SD	9.3 ± 5.8	6.8 ± 4.3	6.8 ± 4.4	< 0.001^3^

*AD* Alzheimer’s disease, *SD* standard deviation, *MMSE* mini mental state examination, *CDR-SB* clinical dementia rating-sum of boxes

^1^*p* Value is derived from Mann–Whitney *U* test

^2^*p* Value is derived from Fisher’s exact test

^3^After adjustment for age and disease duration in a generalized linear model

## Results

### Participants

We identified a total of 20,745 individuals with comprehensive clinical data at baseline (March 2016 NACC data freeze). Of these, 10,527 were diagnosed with AD and 10,218 were cognitively asymptomatic controls. 498 of the individuals with AD were classified, as described in the methods section, as pre-symptomatic, 43 as impaired-not-MCI, 859 as MCI, and 9127 as demented (Fig. 1). Baseline characteristics of these subpopulations are shown in Table 1.

### Seizure recurrence risk

At baseline 138 AD dementia patients with active seizures were identified. After a mean of 7.46 months, 60 of them presented for follow-up (corresponding to a dropout rate of 56.5%), and for 54 of these 60 data for seizures were available. Out of these 54 individuals, 38 still suffered from active seizures. These figures result in a risk for seizure recurrence of 70.4% within 7.46 months.

### Seizures, cognitive performance and age of AD onset

**Fig. 2:**
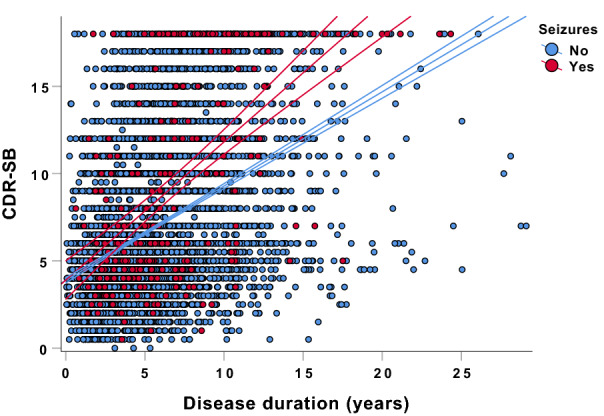
Grouped scatter plot depicting CDR-SB as a function of the presence of a history of seizures and disease duration. There was a highly significant effect of the presence of a history of seizures on CDR-SB towards abnormal over disease duration. Middle lines represent the linear fits of the data, upper and lower lines represent 95% confidence intervals. *CDR-SB* clinical dementia rating-sum of boxes

With respect to cognition and function, AD dementia patients with a history of seizures performed worse in MMSE as well as in CDR-SB compared to those without (mean MMSE score: 16.6 vs. 19.6, *p* < 0.001; mean CDR-SB score: 9.3 vs. 6.8, *p* < 0.001) after adjustment for disease duration and age (Table 2). There was a highly significant effect of a positive history of seizures on the CDR-SB towards abnormal over disease duration (history of seizures*disease duration: *F* = 18.66; *p* < 0.001) (Fig. 2). The age of onset of cognitive symptoms was earlier in AD dementia patients with seizures compared to those without (64.7 vs. 70.4 years, *p* < 0.001).

### Disease duration and seizure prevalence

**Fig. 3:**
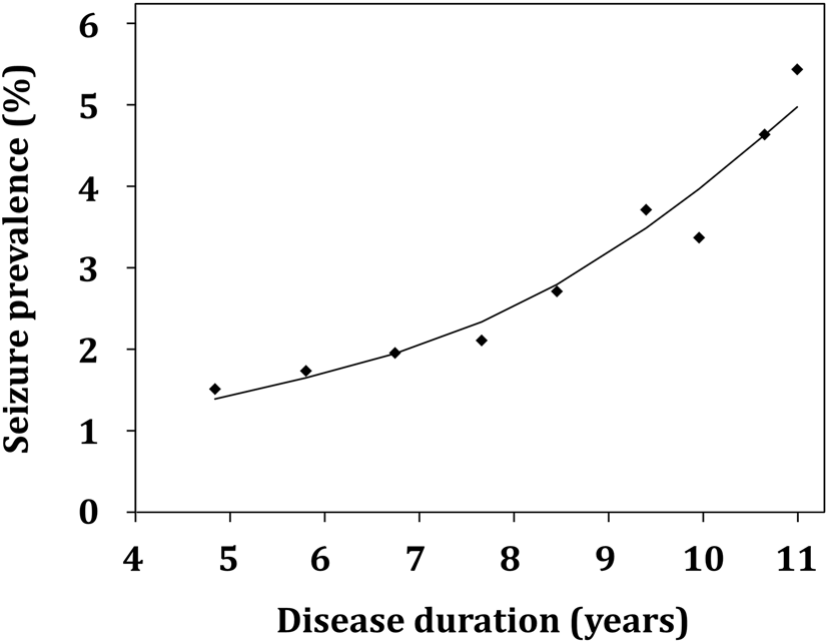
Association of prevalence of active seizures and disease duration in patients with Alzheimer’s disease dementia. Logistic regression with disease duration squared transformed and standardized as explanatory variable (Quadratic equation: logit{*P*(“having a seizure”|disease duration = *d*)} = − 3.56 + 0.44 × (*d−m*_d_/sd_d_)^2^ with *m*_d_ = 72.7 and sd_d_ = 35.0 being the mean and the standard deviation over all disease durations). The effect of disease duration on seizure prevalence was highly significant in patients with AD dementia (OR = 1.55, 95% CI = 1.39–1.73, *p* < 0.001)

The effect of disease duration on seizure prevalence was highly significant in patients with AD dementia (standardized OR = 1.55, 95% CI = 1.39–1.73, *p* < 0.001). The frequency of active seizures amounted to 1.51% after 4.8 years of AD dementia duration and increased to 5.43% at 11.0 years of disease course. On average, prevalence of active seizures in AD dementia rose by 0.64% per year of disease duration (Fig. 3).

### Seizure prevalence

**Fig. 4:**
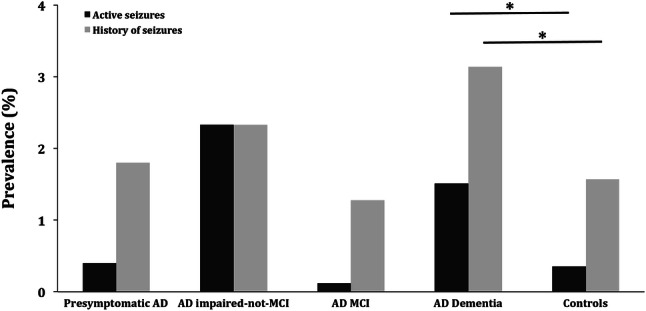
Prevalence of seizures in the various stages of Alzheimer’s disease and in controls. Prevalence of active seizures and a history of seizures were higher in AD dementia patients compared to controls. No differences in seizure prevalence in pre-dementia stages of AD compared to controls were found. *AD* Alzheimer’s disease, *MCI* mild cognitive impairment. *p < 0.001

Active seizures were significantly more prevalent in patients with AD dementia than in controls (1.51% vs. 0.35%, OR = 4.34, 95% CI = 3.01–6.27, *p* < 0.001). Further, AD dementia patients revealed a history of seizures at a higher frequency compared to controls (3.14% vs. 1.57%, OR = 2.03, 95% CI = 1.67–2.46, *p* < 0.001) (Fig. 4).

No differences in the prevalence of active seizures were found in individuals with pre-symptomatic AD (0.4%, *p* = 0.70), AD impaired-not-MCI (2.33%, *p* = 0.14) and AD MCI (0.12%, *p* = 0.36) compared to controls. With respect to a history of seizures, no differences between these groups were apparent (pre-symptomatic AD: 1.8%, *p* = 0.71; AD impaired-not-MCI: 2.33%, *p* = 0.50; AD MCI: 1.28%, *p* = 0.67).

## Discussion

In our analyses of the NACC database with nearly twenty-one thousand individuals, standardized assessment of seizures with information obtained from study participants and their mandatory co-participants, from medical records and from observation, as well as AD diagnosis according to established criteria [13, 14], we revealed for the first time the exceptionally high recurrence risk for seizures of over 70% within about seven and half months in AD dementia patients. Based on this finding, it can be discussed that one unprovoked or reflex seizure should lead to the diagnosis of epilepsy in AD dementia according to the current guidelines of the International League Against Epilepsy (ILAE), which postulates a recurrence risk of at least 60% in ten years after a first unprovoked or reflex seizure for the diagnosis of epilepsy [17]. If AD dementia-associated epilepsy is diagnosed, the high recurrence risk as shown in our study should implicate the consideration of an antiepileptic treatment. It should also be noted, that the recurrence risk may be underestimated in this study as active seizures may prevent AD patients from further study participation. Additionally, a part of the evaluated individuals may already receive an antiepileptic medication mitigating seizure prevalence at follow-up. Further arguments for the consideration of an antiepileptic drug treatment are that AD-associated seizures are responsive to a low dose monotherapy in many cases [17–21], and are associated with poor cognition and function as shown in this study as well as can accelerate cognitive decline [22, 23].

Of particular interest is the question if seizure recurrence risk is higher in AD than in older individuals with epilepsy without an overt neurodegenerative condition. In a recent study a recurrence risk within one year after a first unprovoked seizure of 53% in older individuals (mean age: 73 years; AD dementia patients with seizures in this study for comparison: 71 years) regardless of seizure etiology was reported [24]. These 53% within one year compare to the > 70% within seven and a half months in AD-associated epilepsy as found in this study. An interpretation could be that AD-associated epilepsy features a higher recurrence risk than seizures in older individuals with mixed seizure etiologies. However, it has to be considered that diagnosing of seizures in AD faces particular challenges. On the one hand, seizures can go unrecognized in AD, i.e., due to memory problems of the respective patients. On the other hand, seizures can be wrongly suspected on the basis of cognitive fluctuations and other non-epileptic symptoms.

We found an association of seizures with worse cognitive and functional performance in AD dementia patients. This association may suggest that seizures promote cognitive decline in AD. Another interpretation could be that AD patients who are more severely affected by cognitive deterioration are at an increased risk of seizures. However, we showed that AD patients with seizures performed worse in cognitive and functional tasks compared to patients without seizures after accounting for disease duration as a potential surrogate parameter of disease severity. This may also implicate the consideration of an antiepileptic treatment on an individual basis.

It is still unclear, if seizures are associated with an earlier age of AD onset [5]. The inverse association between prevalence of seizures and age of symptom onset of AD observed in this study may shed light on this lack of clarity. Our finding allows two interpretations. First, seizures may foster AD pathology [25, 26] and precipitate cognitive symptoms [22, 23]. Second, individuals with AD at a younger age are more susceptible to seizures, i.e., feature a lower seizure threshold, compared to older individuals.

With respect to an association between AD severity and seizure risk, findings are at variance in the literature. Some studies suggest such an association [27, 28], whereas others showed an increased risk for seizures in AD that was independent from the disease stage [29, 30]. Adding to clarification, we found a strong association between duration of AD, i.e., the time from the onset of the first cognitive symptom, and the frequency of active seizures.

We confirmed and refined the increased risk for seizures in patients with AD dementia that was suggested previously [4, 5, 31] in a large prospectively assessed dataset with AD diagnosis according to established criteria [13, 14] and standardized seizure assessment. The odds ratio of 4.34 for active seizures found fits well with the estimated two- to six-fold increase in seizure risk in AD patients of a recent review article [5]. Of note, the risk derived from the current analysis is more likely underestimated as seizures in the NACC-UDS are assessed by only a single item [10]. Our results do not indicate that the predementia stage of AD carries an increased risk for seizures. This is particularly remarkable as we stipulated the diagnosis of AD dementia at a subsequent visit for the classification of the predementia stages to improve diagnostic accuracy. This finding is at odds with an increased seizure prevalence in the pre-symptomatic phase of autosomal dominant Alzheimer’s disease (ADAD) [7]. A potential explanation could be that pathology in sporadic AD has to reach a higher threshold to increase seizure risk than in ADAD due to a higher susceptibility of the younger ADAD population for seizures. Another reason could be that the early pre-symptomatic seizures in ADAD are driven by mutation specific effects. However, also a reduced probing of raters in non-demented individuals in this study could be critical.

Both amyloid β and tau, the neuropathological hallmarks of AD, have been linked to seizure generation [5]. Mouse studies suggested a direct excitatory effect of amyloid β on brain networks [32]. Our study showed an increased risk for seizures not before the dementia state of AD and a growing risk with disease duration. As tau pathology seems to be closely linked to the occurrence of cognitive symptoms [33], our findings could be in line with an obligatory presence of both amyloid β and tau for seizure development and further could be in accordance with a proposed synergistic effect of these pathologies on the generation of seizures [34].

Strengths of our study are the large size of the cohort with over twenty thousand individuals in this investigation, yearly prospective data collection over more than a decade, and the standardized assessment with the well-validated UDS [10, 11]. Limitations are that antiepileptic medication which can have an effect on seizure recurrence is not analyzed, semiology of seizures was not assessed, and with respect to the analysis of seizure recurrence risk the dropout rate of approximately 56%. It can be discussed that the dropout rate may in part be caused by ongoing seizures restraining patients from further study participation. Further, the diagnosis of seizures in older individuals is challenging and their outcomes rely on self-report. Stroke history is not evaluated and therefore potential effects of strokes that did not prevent the affected participant from further study participation on seizure occurrence in our study population cannot be estimated precisely. As neuroimaging is not assessed, cortical lesions such as microhemorrhages or subclinical infarcts that could be contributing to the development of seizures cannot be ruled out. Additionally, there could be individuals with ADAD in the population of this study who are known to be at risk for seizures [36]. As the frequency of ADAD causing mutations in patients with AD is about or less than one percent [35] and the mean age of individuals with AD in this study (symptomatic AD: ~ 75 years, presymptomatic AD: ~ 80 years) corroborates the assumption that there is no substantial portion of individuals with ADAD in the study population, we did not perform analyses in detail regarding this subgroup that is expected to be very small.

In summary, in this study we reveal an exceptionally high recurrence risk of seizures in patients with AD dementia that should be considered in treatment of AD-associated seizures. Our results may answer open questions regarding an association of seizures with lower cognitive performance and a younger age of onset. A strong association between disease duration and seizure risk is demonstrated. Furthermore, the evidence for an increased seizure risk in AD dementia is confirmed and refined in a large cohort with consistently, prospectively assessed data.

Our findings corroborate the relevance and impact of seizures in clinical care of AD dementia, inform individual treatment decisions, and emphasize the necessity of prospective, double-blind, randomized, controlled, parallel-group antiepileptic treatment trials in AD.
